# Assessing the prevalence of non-medical prescription opioid use in the Canadian general adult population: evidence of large variation depending on survey questions used

**DOI:** 10.1186/1471-244X-13-6

**Published:** 2013-01-04

**Authors:** Kevin D Shield, Anca Ialomiteanu, Benedikt Fischer, Jürgen Rehm

**Affiliations:** 1 Centre for Addiction and Mental Health (CAMH), 33 Russell Street, Toronto, Ontario, M5S 2S1, Canada; 2 Institute of Medical Sciences (IMS), University of Toronto, Toronto,, Ontario, Canada; 3 Dalla Lana School of Public Health (DLSPH), University of Toronto, Toronto, Ontario, Canada; 4 Centre for Applied Research in Mental Health and Addiction, Simon Fraser University, Faculty of Health Sciences, Vancouver, British Columbia, Canada; 5 Institute for Clinical Psychology and Psychotherapy, Technische Universität Dresden, Chemnitzer Str. 46, Dresden, Germany

**Keywords:** Prescription Opioids, Non-medical use, Surveillance, General population, Morbidity

## Abstract

**Background:**

Morbidity and mortality related to Prescription Opioid Analgesics (POAs) have been rising sharply in North America. Non-Medical Prescription Opioid Use (NMPOU) in the general population is a key indicator of POA-related harm, yet the role of question item design for best NMPOU prevalence estimates in general population surveys is unclear, and existing NMPOU survey data for Canada are limited.

**Methods:**

We tested the impact of different NMPOU question items by comparing an item in the 2008 and 2009 (N = 2,017) samples of the CAMH Monitor surveys – an Ontario adult general population survey – with a newly developed item used in the 2010 (N = 2,015) samples of the Centre for Addiction and Mental Health (CAMH) Monitor surveys. To control for a potential difference in the population demographics between surveys, we adjusted for gender, age, region, income, prescription opioid use, cigarette smoking, weekly binge drinking, cannabis use in the past three months, and psychological distress in our analyses.

**Results:**

The prevalence of NMPOU as measured by the 2008 and 2009 CAMH monitor (2.0% [95% CI: 1.2% to 2.8%]) was significantly different when compared to the prevalence of NMPOU as measured by the 2010 CAMH monitor (7.7% [95% CI: 6.3% to 9.2%]) (p < 0.001). This difference was also found when stratifying our analysis by sex (p < 0.001) and when adjusting for all potential confounding covariates.

**Conclusion:**

It is highly unlikely that the extensive NMPOU prevalence differences observed from the different survey items reflect an actual increase of NMPOU or changes in NMPOU determinants, but rather point to measurement effects. It appears that we currently do not have accurate estimates of NMPOU in the Canadian general population, even though these estimates are needed to guide and implement targeted interventions. Given the current substantial morbidity and mortality impact of NMPOU, there is an urgent need to systematically develop, validate and standardize NMPOU items for future general population surveys in Canada.

## Background

In Canada and the United States (U.S.), levels of Prescription Opioid Analgesic (POA) use have been rising since 2000; globally, Canada and the U.S. have the highest levels of POA use [1]. In this context, POA-related morbidity and mortality levels have also increased in recent years [2-6], constituting a major public health problem.

A key epidemiological indicator of POA-related problems is the prevalence of Non-Medical Prescription Opioid Use (NMPOU), which is defined as using POAs when they are not prescribed by a physician, or using POAs for purposes other than as prescribed; the definition of NMPOU excludes the underuse of POAs. The prevalence of NMPOU is strongly correlated to levels of POA-related morbidity and mortality [6], and hence the prevalence of NMPOU in the general population needs to be accurately identified for targeted preventive interventions [6-9].

NMPOU, can be defined and operationalized in different ways. For example, the Canadian Alcohol and Other Drug Use Monitoring Survey (CADUMS) initially surveyed NMPOU in the Canadian general adult population (15+ years) in 2008 based on the following questions: 1) “During the past 12 months, did you ever use pain relievers for the feelings [they] caused or to get high?”, and/or 2) Were the pain relievers used during the past 12 months obtained from i) “a prescription written for someone else such as a family member or a friend,” ii) “bought from someone else, without a prescription,” iii) “from any other source” [10]. On this basis, the CADUMS found an NMPOU prevalence of 0.4%, which amounted to about one-tenth of the NMPOU prevalence of 4.8% estimated for the United States (U.S.) general youth and adult (12+ years) population in 2008 by the National Survey on Drug Use and Health (NSDUH) [11,12].

In contrast to the CADUMS, NMPOU is defined in the NSDUH as having provided the answer “yes” to the question: “Have you ever, even once, used (name of prescription opioid) that was not prescribed for you or that you took only for the experience or feeling it caused?” [12]. Given the amount of POAs that are prescribed in Canada and in the U.S., and the association between `POA use and NMPOU [13], NMPOU prevalence estimates for Canada should be approximately half the prevalence of NMPOU in the U.S. Differences in the prevalence estimates of NMPOU obtained from the CADUMS and from the NSDUH may be due in part to variations in the formulation of questions asked in each of these surveys which result in differing responses. As measurement differences in NMPOU have not been investigated, there is a timely need and opportunity to investigate estimate variations that may arise from differences in how NMPOU is operationalized.

## Methods

To examine potential measurement effects for assessing the prevalence of NMPOU in Canada, we tested an alternative NMPOU item constructs in the Centre for Addiction and Mental Health (CAMH) Monitor surveys [14,15].The 2008, 2009 and 2010 CAMH Monitor surveys employed a two-stage, probability sample based, computer-assisted telephone interviewing, random-digit dialing method, and sampled the Ontario general adult population (18+ years). Each month of the survey a sampling frame of all active area codes and exchanges in Ontario was obtained from ATI long lines tape. Telephone numbers from this source, as well as telephone numbers between or on either side of listed numbers were included in the sampling frame. This method allowed for any person with a home phone (listed or unlisted) or a cellphone to be included in the sampling frame; however, a proportion of people with an unlisted number and/or a cellphone were not included in the sampling frame. The sampling frame was then stratified into regions (based on counties) as follows: Toronto, Central West Ontario, Central East Ontario, West Ontario, East Ontario, and North Ontario (see [14-16] for the exact counties included within each region). Within each stratum, a random sample of telephone numbers was selected with equal probability. To increase the response rate in the stratum of Toronto, a letter was sent to the selected household informing them that they would be contacted and describing the history and objectives of the CAMH Monitor survey. Within selected households, the respondent who was 18 years of age or older, who had the most recent birthday of the eligible household members, and who could complete the interview in either English or French was selected to participate in the survey. A minimum of 12 call-backs were placed to unanswered numbers and all households who refused to participate on the first contact were re-contacted and asked again to participate. No incentive was offered for participation. The procedures and interviews of each cycle of the CAMH Monitor surveys were approved by the CAMH Research Ethics Board [14,15].

NMPOU questions in the 2008 and 2009 waves of the CAMH Monitor surveys were asked from July to December, whereas the 2010 NMPOU data were collected from January to December of the respective year. Response rates for the CAMH Monitor surveys were calculated using the method proposed by the American Association for Public Opinion Research (response rate calculation #3) which takes into account in the response rate calculation the number of telephone numbers which may be eligible to be included in the survey [14-16]. This formula for calculating the response rate is as follows:

(1) $$\mathrm{RR}=\frac{I}{\left(I+P\right)+\left(R+\mathrm{NC}\right)+e\left(\mathrm{UH}\right)}$$

where I represents the number of completions, P represents the number of partial completions, R represents the refusals/breakoffs, NC represents non-contacts, e represents the estimated proportion of cases of unknown eligibility that are eligible, and UH represents the number of telephone numbers which may be eligible to be included in the survey.

In the 2008, 2009 and 2010 waves of the CAMH Monitor surveys POAs were defined as “pain relievers that are obtained by a prescription from a doctor or dentist such as Percocet, Percodan, Demerol, OxyContin, Tylenol #3 or other products or pain relievers with codeine that are obtained in a pharmacy. Some people use these medications to treat pain resulting from an illness, injury.” NMPOU in the 2008 and 2009 waves of the CAMH Monitor surveys was measured similarly to the CADUMS questions described above as follows: 1) “Thinking about all the pain relievers you have used during the past 12 months did you get any of them” i) “from a prescription written for someone else such as a family member or a friend”, ii) “bought from someone else, without a prescription” and/or iii) “from any other source (defined as a source other than the previously mentioned sources and a prescription written for you),” and 2) “During the past 12 months, did you ever use pain relievers for the feelings it caused or to get high?” The alternative NMPOU item tested in the 2010 wave of the CAMH Monitor survey was based on the question: “In the past 12 months how many times, if at all, have you used any such pain relievers without a prescription or without a doctor telling you to take them?”

The items used in the 2008 and 2009 waves of the CAMH Monitor surveys were initially pretested with 25 respondents in November 2007 and included as a pilot study in December of the 2007 wave of the CAMH Monitor survey (N = 175). Applicable questions were asked at the end of the POA items in the 2009 cycle of the CAMH Monitor survey to evaluate the response process; 96.4% of respondents declared that the questions on POA use were not difficult to understand, and 96.1% of respondents did not need the questions to be repeated to them before answering [17]. The items used in the 2010 wave of the CAMH Monitor survey were also initially asked in the 2007 and the 2009 cycles of the Ontario Student Drug Use and Health Survey. These questions were revised for the 2010 wave of the CAMH Monitor survey based on pretesting which confirmed item comprehension and feasibility.

For our analysis of the difference in the measurement effect of the alternative NMPOU survey items on the estimated prevalence of NMPOU, we controlled for the following demographic variables: age (grouped into three categories: 18–29, 30–54, 55+), region (living in Toronto, the rest of Ontario), and household income (<$30,000, $30,000–79,000, $80,000+, not stated). The substance use variables we controlled for in our analysis were tobacco use (defined as either daily or occasional (during the last 12 months) cigarette smoking), weekly binge drinking (defined as drinking five or more drinks on one occasion at least once a week in the previous 12 months), and cannabis use (defined as using cannabis at least once in the previous 12 months).

We also controlled for psychological distress, as measured by the 12-item General Health Questionnaire (GHQ-12). The GHQ-12 is a screening instrument that evaluates depression/anxiety and problems with social functioning [18] and we used a cut-off score of 3 or more on the GHQ-12 as an indication of elevated psychological distress.

All statistical analyses were performed using population expansion weights. Population expansion weights were constructed using census data from Statistics Canada on the number of people living in each stratum by age (using the age categories of 18-24, 25-44, 45-64, 65+) and by sex [14-16]. Significant differences between prevalence estimates were calculated using chi-square tests with a second-order Rao-Scott adjustment for survey data for the unadjusted analysis [19]. For the unadjusted and adjusted regression analyses, we employed a general linear model adjusted for survey design. All statistics were performed using the statistical software package R version 2.15.1 [20], and the statistical software package Survey (a statistical package for R) [21].

## Results

The CAMH Monitor survey response rates were 56.6% for July to December in 2008, 57.5% for July to December in 2009, and 50.6% for January to December in 2010. The NMPOU items run in the 2008 and 2009 waves of the CAMH Monitor surveys had valid data for n = 1,021 (effective response rate of 56.1%) for 2008 and n = 996 (effective response rate of 57.1%) for 2009 respectively. The NMPOU item run in the 2010 wave of the CAMH Monitor survey had valid data for n = 2,015 (effective response rate of 50.2%). The response rates for the 2008, 2009 and 2010 waves of the CAMH Monitor surveys did not vary significantly by month.

Table 1 outlines the prevalence estimates for the different NMPOU items run in the 2008 and 2009 CAMH Monitor survey samples and in the 2010 CAMH Monitor survey sample. Table 2 outlines the prevalence of NMPOU by gender. The prevalence of NMPOU in Ontario in 2008 and 2009 as measured by the 2008 and 2009 waves of the CAMH Monitor surveys was 2.0% (95% Confidence Interval (CI): 1.2% to 2.8%) (2.3% for 2008 and 1.7% for 2009), and was 7.7% (95% CI: 6.3% to 9.2%) in 2010 as measured by the 2010 wave of the CAMH Monitor survey. This difference in the measured prevalence of NMPOU in the 2008 and 2009 CAMH Monitor surveys and in the 2010 CAMH Monitor survey was significant (p <0.001). Significant differences between the NMPOU items were also observed when the analysis was stratified by sex (p <0.001 for women and p <0.001 for men). The Additional file 1: Web appendix outlines these prevalence estimates using no weights.

**Table 1:** Characteristics of Ontario adults (18+ years) as measured by the 2008, 2009 and 2010 CAMH Monitor surveys

	**CAMH Monitor 2008 and 2009**			**CAMH Monitor 2010**		
	**n**	**Point estimate**	**95% Confidence intervals**	**n**	**Point estimate**	**95% Confidence intervals**
Gender						
Men	896	50.5%	(47.8% to 53.2%)	887	49.8%	(47.1% to 52.5%)
Women	1134	49.5%	(46.8% to 52.2%)	1137	50.2%	(47.5% to 52.9%)
Age (years)						
18–29	295	26.7%	(23.8% to 29.5%)	319	28.2%	(25.4% to 31.1%)
30–54	826	43.1%	(40.4% to 45.8%)	756	39.6%	(36.0% to 42.3%)
55+	844	30.2%	(28.0% to 32.5%)	912	32.1%	(29.8% to 34.4%)
Region						
Toronto	317	21.1%	(19.2% to 23.0%)	332	21.9%	(19.9% to 23.8%)
Rest of Ontario	1713	78.9%	(77.0% to 80.8%)	1692	78.1%	(76.2% to 80.1%)
Income						
<$30,000	250	8.5%	(7.1% to 9.9%)	242	8.7%	(7.2% to 10.1%)
$30,000 to $49,999	254	10.4%	(8.9% to 11.9%)	269	10.4%	(9.0% to 11.9%)
$50,000 to $79,999	390	19.2%	(17.2% to 21.3%)	370	17.7%	(15.7% to 19.7%)
$80,000+	619	35.0%	(32.4% to 37.6%)	670	38.7%	(36.1% to 41.4%)
Not stated	517	26.9%	(24.4% to 29.3%)	473	24.5%	(22.1% to 26.8%)
Prescription opioid use						
Yes	475	21.3%	(19.1% to 23.4%)	485	23.1%	(20.8% to 25.3%)
No	1543	78.7%	(76.6% to 80.9%)	1515	76.9%	(74.7% to 79.2%)
Non-medical prescription opioid use						
Yes	40	2.0%	(1.2% to 2.8%)	143	7.7%	(6.3% to 9.2%)
No	1977	98.0%	(97.2% to 98.8%)	1872	92.3%	(90.8% to 93.7%)
Cigarette smoking						
Current	399	18.9%	(16.8% to 21.0%)	373	19.4%	(17.2% to 21.6%)
Former	654	28.2%	(25.9% to 30.4%)	654	28.2%	(25.9% to 30.6%)
Never smoker	973	52.9%	(50.2% to 55.6%)	991	52.4%	(49.7% to 55.1%)
Weekly binge drinking						
Yes	133	7.9%	(6.4% to 9.4%)	144	8.2%	(6.7% to 9.8%)
No	1883	92.1%	(90.6% to 93.6%)	1866	91.8%	(90.2% to 93.3%)
Cannabis use (past 3 months)						
Yes	148	10.1%	(8.3% to 12.0%)	186	12.0%	(10.0% to 14.0%)
No	1876	89.9%	(88.0% to 91.7%)	1838	88.0%	(86.0% to 90.0%)
Psychological distress (GHQ 3+)						
Yes	284	13.4%	(11.5% to 15.2%)	277	14.6%	(12.6% to 16.6%)
No	1743	86.6%	(84.8% to 88.5%)	1746	85.4%	(83.4% to 87.4%)

**Table 2:** Characteristics of Ontario adults (18+ years) as measured by the 2008, 2009 and 2010 CAMH Monitor surveys by gender

	**Women**						**Men**					
	**CAMH Monitor 2008 and 2009**			**CAMH Monitor 2010**			**CAMH Monitor 2008 and 2009**			**CAMH Monitor 2010**		
	**n**	**Point estimate**	**95% Confidence intervals**	**n**	**Point estimate**	**95% Confidence intervals**	**n**	**Point estimate**	**95% Confidence intervals**	**n**	**Point estimate**	**95% Confidence intervals**
Age (years)												
18–29	147	26.2%	(22.1% to 30.2%)	157	24.5%	(21.0% to 28.0%)	148	27.1%	(23.2% to 31.1%)	162	32.0%	(27.7% to 36.3%)
30–54	454	42.9%	(39.2% to 46.6%)	414	40.5%	(37.0% to 44.0%)	372	43.3%	(39.4% to 47.2%)	342	38.8%	(34.9% to 42.6%)
55+	488	30.9%	(27.9% to 34.0%)	545	35.0%	(31.8% to 38.1%)	356	43.3%	(40.0% to 46.6%)	367	38.8%	(35.5% to 42.0%)
Region												
Toronto	179	21.7%	(18.7% to 24.8%)	196	23.6%	(20.7% to 26.5%)	138	20.5%	(17.4% to 23.6%)	136	20.1%	(16.9% to 23.4%)
Rest of Ontario	955	78.3%	(75.2% to 81.3%)	941	76.4%	(73.5% to 79.3%)	758	79.5%	(76.4% to 82.6%)	751	79.9%	(76.6% to 83.1%)
Income												
<$30,000	163	9.8%	(7.9% to 11.7%)	156	9.5%	(7.7% to 11.4%)	87	7.2%	(5.3% to 9.2%)	86	7.8%	(5.6% to 10.0%)
$30,000 to $49,999	139	10.1%	(8.1% to 12.1%)	164	11.4%	(9.3% to 13.4%)	115	10.7%	(8.4% to 12.9%)	105	9.5%	(7.4% to 11.6%)
$50,000 to $79,999	216	19.5%	(16.7% to 22.3%)	189	16.7%	(14.1% to 19.3%)	174	19.0%	(16.0% to 12.9%)	181	18.7%	(15.7% to 21.8%)
$80,000+	290	30.7%	(27.2% to 34.3%)	336	35.3%	(31.8% to 38.7%)	329	19.0%	(15.2% to 22.7%)	334	18.7%	(14.7% to 22.7%)
Not stated	326	29.9%	(26.4% to 33.3%)	292	27.1%	(23.9% to 30.4%)	191	23.9%	(20.4% to 27.4%)	181	21.8%	(18.3% to 25.2%)
Prescription opioid use												
Yes	279	22.7%	(19.6% to 25.7%)	293	24.7%	(21.7% to 27.7%)	196	19.9%	(16.9% to 22.9%)	192	21.4%	(18.1% to 24.8%)
No	848	77.3%	(74.3% to 80.4%)	829	75.3%	(72.3% to 78.3%)	695	80.1%	(77.1% to 83.1%)	686	78.6%	(75.2% to 81.9%)
Non-medical prescription opioid use												
Yes	20	1.6%	(0.8% to 2.5%)	77	7.4%	(5.5% to 9.3%)	20	2.4%	(1.0% to 3.7%)	66	8.1%	(5.9% to 10.3%)
No	1106	98.4%	(97.5% to 99.2%)	1058	92.6%	(90.7% to 94.5%)	871	97.6%	(96.3% to 99.0%)	814	91.9%	(89.7% to 94.1%)
Cigarette smoking												
Current	194	15.2%	(12.6% to 17.7%)	188	15.7%	(13.1% to 18.2%)	205	22.6%	(19.3% to 25.9%)	185	23.1%	(19.6% to 26.7%)
Former	342	25.9%	(22.9% to 28.9%)	340	26.1%	(23.1% to 29.0%)	312	30.4%	(27.0% to 33.8%)	314	30.4%	(26.9% to 34.0%)
Never smoker	595	59.0%	(55.4% to 62.5%)	607	58.3%	(54.8% to 61.7%)	378	47.0%	(43.0% to 50.9%)	384	46.4%	(42.4% to 50.5%)
Weekly binge drinking												
Yes	27	2.9%	(1.5% to 4.4%)	29	3.3%	(1.9% to 4.6%)	106	12.8%	(10.2% to 15.5%)	115	13.2%	(10.4% to 16.0%)
No	1102	97.1%	(95.6% to 98.5%)	1102	96.7%	(95.4% to 98.1%)	781	87.2%	(84.5% to 89.8%)	764	86.8%	(84.0% to 89.6%)
Cannabis use (past 3 months)												
Yes	46	6.1%	(4.0% to 8.2%)	61	6.3%	(4.5% to 8.2%)	102	14.1%	(11.1% to 17.0%)	125	17.7%	(14.2% to 21.1%)
No	1084	93.9%	(91.8% to 96.0%)	1076	93.7%	(91.8% to 95.5%)	792	85.9%	(83.0% to 88.9%)	762	82.3%	(78.9% to 85.8%)
Psychological distress (GHQ 3+)												
Yes	178	16.3%	(13.5% to 19.1%)	180	16.8%	(14.0% to 19.5%)	106	10.5%	(8.2% to 12.9%)	97	12.4%	(9.5% to 15.3%)
No	954	83.7%	(80.9% to 86.5%)	957	83.2%	(80.5% to 86.0%)	789	89.5%	(87.1% to 91.8%)	789	87.6%	(84.7% to 90.5%)

Table 3 outlines the unadjusted and adjusted odds ratios for NMPOU by survey and gender. When comparing the 2008 and 2009 waves to the 2010 wave of the CAMH Monitor surveys, the odds of NMPOU increased by 3.99 (95% CI: 2.52 to 6.31) when not adjusting for any covariates, and by 4.12 (95% CI: 2.66 to 6.37) when adjusting for all confounding covariates. The interaction between gender and survey year on the odds of NMPOU was not found to be significant (p > 0.10).

**Table 3:** Odds ratios of non-medical prescription opioid use as measured by the 2010 CAMH Monitor survey compared to the 2008 and 2009 CAMH Monitor surveys

			**Total**		**Men**		**Women**	
**Outcome**	**Model**	**Survey year**	**Point estimate**	**95% Confidence intervals**	**Point estimate**	**95% Confidence intervals**	**Point estimate**	**95% Confidence intervals**
Non-medical prescription opioid use	Model 1 (Unadjusted)	2010	3.99	(2.52 to 6.31)	3.60	(1.89 to 6.88)	4.58	(2.46 to 8.54)
		2008 and 2009	REF	-	REF	-	REF	-
	Model 2 (Adjusted)	2010	4.12	(2.66 to 6.37)	3.96	(2.27 to 6.93)	4.90	(2.69 to 8.95)
		2008 and 2009	REF	-	REF	-	REF	-

Model 2 was adjusted for the following variables: gender (only for the regression including both men and women), age, region, income, prescription opioid use, cigarette smoking, weekly binge drinking, cannabis use (past three months) and psychological distress.

## Discussion

Our results suggest that NMPOU survey estimates differ markedly depending on the question items used. The estimated prevalence of NMPOU in Ontario is approximately 210,000 people (derived from population estimates of Ontario in 2010) when based on the estimates from the 2008 and 2009 CAMH Monitor surveys, and 808,700 people (derived from population estimates of Ontario in 2010) when based on the 2010 CAMH Monitor survey[22].

Self-reported non-medical drug use is a subjective concept, and difficult to measure as it is commonly under-reported in surveys, with cognitive models of survey interviews suggesting that reporting errors originate from i) poor comprehension of the questions asked, ii) inaccurate recall of the information, and iii) deliberate misreporting of information [23]. The NMPOU questions in the 2008 and 2009 CAMH Monitor surveys asked about the source and the use of POA, while the NMPOU question in the 2010 CAMH Monitor survey asked the participant whether they used prescription opioids without a prescription or a doctor telling them to do so. Additionally, some of the NMPOU items may deal with the illicit use of drugs, a socially disapproved of and unlawful behaviour, which may have led participants in the 2008 and 2009 CAMH Monitor surveys to edit their responses [23-25].

### Limitations

It is important to note that NMPOU may have actually increased and/or the determinants of NMPOU may have changed between 2008, 2009, and 2010, although it is highly unlikely that the entirety of the observed differences in NMPOU prevalence can be explained by these factors [26]. When comparing the difference in the NMPOU prevalence in the CAMH Monitor surveys to the U.S. data for trends in the prevalence of NMPOU in the same three years (4.8% for 2008, 4.9% for 2009 and 4.8% for 2010 [11,12,27]), it seems unrealistic to suggest that the difference found in our study between 2008/2009 and 2010 is completely attributable to such a major increase in NMPOU. Additionally, to control for a potential change in the determinants of NMPOU, we performed a regression analysis that controlled for the covariates of gender (only for the regression that included both men and women), age, region, income, prescription opioid use, cigarette smoking, weekly binge drinking, cannabis use in the past three months, and psychological distress, which have been shown to be associated with NMPOU [28]. Thus, the observed difference in the prevalence estimates for 2008/2009 versus 2010 is likely due to measurement error.

Both prevalence estimates of NMPOU are also biased by factors other than the question used to measure NMPOU, and thus are likely underestimates of NMPOU in Ontario. The CAMH Monitor was a telephone-based population survey and excluded people who did not own a phone. This excluded group includes homeless individuals who have been shown to be more likely to use prescription opioids than the general popula-tion [29], thereby leading to a probable underestimation of the prevalence of NMPOU. In addition to excluding people who do not have a phone, the surveys also excluded people who did not speak either English or French; however, there were very few people excluded from the 2008, 2009, or 2010 CAMH Monitor surveys because they did not speak either English or French [14-16]. An additional difficulty with telephone surveys is that those people who are unlikely to respond are more likely to engage in habits that are detrimental to their health [30]. To obtain accurate estimates of NMPOU in Ontario, both the question item constructs and protocols used to calculate these prevalence estimates need to be improved.

## Conclusions

The results of this study demonstrate that we cannot be certain to have a reliable or valid general population survey estimates of the number of individuals engaging in NMPOU in Canada. Thus, validity studies investigating the effects of NMPOU items on a study participant’s comprehension, interpretation, information retrieval, judgment formation, and response editing, are needed to validate current measures and to create improved, standardized measures of NMPOU in order to generate more reliable or valid estimates of the number of individuals engaging in NMPOU in Canada.

In summary, given the morbidity and mortality impact of NMPOU and the requirement for evidence-based targeted interventions, there is an urgent need to systematically develop, validate, and standardize NMPOU items for future general population surveys [31].

## Abbreviations

POA: Prescription opioid analgesic; NMPOU: Non-medical prescription opioid use; CADUMS: Canadian Alcohol and Other Drug Use Monitoring Survey.

## Competing interests

The authors declare that they have no competing interests.

## Authors’contributions

KS, AI, JR and BF conceptualized the overall article, acquired all data, and contributed to the methodology. KS performed all statistical analyses. KS, AI, JR and BF contributed to the writing of the manuscript and approved the final version. All authors read and approved the final manuscript.

## Pre-publication history

The pre-publication history for this paper can be accessed here:

http://www.biomedcentral.com/1471-244X/13/6/prepub

## Supplementary Material

Additional file 1 **Web Appendix. **Unweigthed prevalence estimates. Click here for file
