# Supplementary material for: Assessing the prevalence of non-medical prescription opioid use in the Canadian general adult population: evidence of large variation depending on survey questions used
Source: BMC Psychiatry. 2013 Jan 4;13:6. doi: 10.1186/1471-244X-13-6 (PMC3546044; doi:10.1186/1471-244X-13-6)
Supplement: Additional file 1 — Web Appendix. Unweigthed prevalence estimates. [file 1471-244X-13-6-S1.docx]

**Web Appendix. Unweigthed prevalence estimates**

Table A1: Characteristics of Ontario adults (18+ years) as measured by the 2008, 2009 and 2010 CAMH Monitor surveys (unweighted estiamtes)

Table A2: Characteristics of Ontario adults (18+ years) as measured by the 2008, 2009 and 2010 CAMH Monitor surveys by gender (unweighted estiamtes)

Table 1: Characteristics of Ontario adults (18+ years) as measured by the 2008, 2009 and 2010 CAMH Monitor surveys (unweighted estiamtes)

|  |  | CAMH Monitor 2008 and 2009 | | |  | CAMH Monitor 2010 | | |
| --- | --- | --- | --- | --- | --- | --- | --- | --- |
|  |  | n | Point estimate | 95% Confidence intervals |  | n | Point estimate | 95% Confidence intervals |
| Gender | |  |  |  |  |  |  |  |
|  | Men | 896 | 44.1% | (42.0% to 46.3%) |  | 887 | 43.8% | (41.6% to 46.0%) |
|  | Women | 1134 | 55.9% | (53.7% to 58.0%) |  | 1137 | 56.2% | (54.0% to 58.4%) |
| Age (years) | |  |  |  |  |  |  |  |
|  | 18-29 | 295 | 15.0% | (13.5% to 16.7%) |  | 319 | 16.1% | (14.5% to 17.7%) |
|  | 30-54 | 826 | 42.0% | (39.8% to 44.3%) |  | 756 | 38.0% | (35.9% to 40.2%) |
|  | 55+ | 844 | 43.0% | (40.7% to 45.2%) |  | 912 | 45.9% | (43.7% to 48.1%) |
| Region | |  |  |  |  |  |  |  |
|  | Toronto | 317 | 15.6% | (14.1% to 17.3%) |  | 332 | 16.4% | (14.8% to 18.1%) |
|  | Rest of Ontario | 1713 | 84.4% | (82.7% to 85.9%) |  | 1692 | 83.6% | (81.9% to 85.2%) |
| Income | |  |  |  |  |  |  |  |
|  | <$30,000 | 250 | 12.3% | (10.9% to 13.8%) |  | 242 | 12.0% | (10.6% to 13.5%) |
|  | $30,000 to $49,999 | 254 | 12.5% | (11.1% to 14.0%) |  | 269 | 13.3% | (11.8% to 14.8%) |
|  | $50,000 to $79,999 | 390 | 19.2% | (17.5% to 21.0%) |  | 370 | 18.3% | (16.6% to 20.0%) |
|  | $80,000+ | 619 | 30.5% | (28.5% to 32.5%) |  | 670 | 33.1% | (31.1% to 35.2%) |
|  | Not stated | 517 | 25.5% | (23.6% to 27.4%) |  | 473 | 23.4% | (21.5% to 25.3%) |
| Prescription opioid use | |  |  |  |  |  |  |  |
|  | Yes | 475 | 23.5% | (21.7% to 25.5%) |  | 485 | 24.3% | (22.4% to 26.2%) |
|  | No | 1543 | 76.5% | (74.5% to 78.3%) |  | 1515 | 75.8% | (73.8% to 77.6%) |
| Non-medical prescription opioid use | |  |  |  |  |  |  |  |
|  | Yes | 40 | 2.0% | (1.4% to 2.7%) |  | 143 | 7.1% | (6.0% to 8.3%) |
|  | No | 1977 | 98.0% | (97.3% to 98.6%) |  | 1872 | 92.9% | (91.7% to 94.0%) |
| Cigarette smoking | |  |  |  |  |  |  |  |
|  | Current | 399 | 19.7% | (18.0% to 21.5%) |  | 373 | 18.5% | (16.8% to 20.2%) |
|  | Former | 654 | 32.3% | (30.2% to 34.4%) |  | 654 | 32.4% | (30.4% to 34.5%) |
|  | Never smoker | 973 | 48.0% | (45.8% to 50.2%) |  | 991 | 49.1% | (46.9% to 51.3%) |
| Weekly binge drinking | |  |  |  |  |  |  |  |
|  | Yes | 133 | 6.6% | (5.6% to 7.8%) |  | 144 | 7.2% | (6.1% to 8.4%) |
|  | No | 1883 | 93.4% | (92.2% to 94.4%) |  | 1866 | 92.8% | (91.6% to 93.9%) |
| Cannabis use (past 3 months) | |  |  |  |  |  |  |  |
|  | Yes | 148 | 7.3% | (6.2% to 8.5%) |  | 186 | 9.2% | (8.0% to 10.5%) |
|  | No | 1876 | 92.7% | (91.5% to 93.8%) |  | 1838 | 90.8% | (89.5% to 92.0%) |
| Psychological distress (GHQ 3+) | |  |  |  |  |  |  |  |
|  | Yes | 284 | 14.0% | (12.5% to 15.6%) |  | 277 | 13.7% | (12.2% to 15.3%) |
|  | No | 1743 | 86.0% | (84.4% to 87.5%) |  | 1746 | 86.3% | (84.7% to 87.8%) |

Table 2: Characteristics of Ontario adults (18+ years) as measured by the 2008, 2009 and 2010 CAMH Monitor surveys by gender (unweighted estiamtes)

|  |  | Women | | | | | | |  | Men | | | | | | |
| --- | --- | --- | --- | --- | --- | --- | --- | --- | --- | --- | --- | --- | --- | --- | --- | --- |
|  |  | CAMH Monitor 2008 and 2009 | | |  | CAMH Monitor 2010 | | |  | CAMH Monitor 2008 and 2009 | | |  | CAMH Monitor 2010 | | |
|  |  | n | Point estimate | 95% Confidence intervals |  | n | Point estimate | 95% Confidence intervals |  | n | Point estimate | 95% Confidence intervals |  | n | Point estimate | 95% Confidence intervals |
| Age (years) | |  |  |  |  |  |  |  |  |  |  |  |  |  |  |  |
|  | 18-29 | 147 | 13.5% | (11.5% to 15.7%) |  | 157 | 14.1% | (12.1% to 16.2%) |  | 148 | 16.9% | (14.5% to 19.5%) |  | 162 | 18.6% | (16.1% to 21.3%) |
|  | 30-54 | 454 | 41.7% | (38.7% to 44.7%) |  | 414 | 37.1% | (34.3% to 40.0%) |  | 372 | 42.5% | (39.2% to 45.8%) |  | 342 | 39.3% | (36.0% to 42.6%) |
|  | 55+ | 488 | 44.8% | (41.8% to 47.8%) |  | 545 | 48.8% | (45.9% to 51.8%) |  | 356 | 40.6% | (37.4% to 44.0%) |  | 367 | 42.1% | (38.8% to 45.5%) |
| Region | |  |  |  |  |  |  |  |  |  |  |  |  |  |  |  |
|  | Toronto | 179 | 15.8% | (13.7% to 18.0%) |  | 196 | 17.2% | (15.1% to 19.6%) |  | 138 | 15.4% | (13.1% to 17.9%) |  | 136 | 15.3% | (13.0% to 17.9%) |
|  | Rest of Ontario | 955 | 84.2% | (82.0% to 86.3%) |  | 941 | 82.8% | (80.4% to 84.9%) |  | 758 | 84.6% | (82.1% to 86.9%) |  | 751 | 84.7% | (82.1% to 87.0%) |
| Income | |  |  |  |  |  |  |  |  |  |  |  |  |  |  |  |
|  | <$30,000 | 163 | 14.4% | (12.4% to 16.6%) |  | 156 | 13.7% | (11.8% to 15.9%) |  | 87 | 9.7% | (7.9% to 11.8%) |  | 86 | 9.7% | (7.8% to 11.8%) |
|  | $30,000 to $49,999 | 139 | 12.3% | (10.4% to 14.3%) |  | 164 | 14.4% | (12.4% to 16.6%) |  | 115 | 12.8% | (10.7% to 15.2%) |  | 105 | 11.8% | (9.8% to 14.1%) |
|  | $50,000 to $79,999 | 216 | 19.0% | (16.8% to 21.5%) |  | 189 | 16.6% | (14.5% to 18.9%) |  | 174 | 19.4% | (16.9% to 22.2%) |  | 181 | 20.4% | (17.8% to 23.2%) |
|  | $80,000+ | 290 | 25.6% | (23.1% to 28.2%) |  | 336 | 29.6% | (26.9% to 32.3%) |  | 329 | 36.7% | (33.6% to 40.0%) |  | 334 | 37.7% | (34.5% to 40.9%) |
|  | Not stated | 326 | 28.7% | (26.1% to 31.5%) |  | 292 | 25.7% | (23.3% to 28.3%) |  | 191 | 21.3% | (18.7% to 24.1%) |  | 181 | 20.4% | (17.8% to 23.2%) |
| Prescription opioid use | |  |  |  |  |  |  |  |  |  |  |  |  |  |  |  |
|  | Yes | 279 | 24.8% | (22.3% to 27.4%) |  | 293 | 26.1% | (23.6% to 28.8%) |  | 196 | 22.0% | (19.3% to 24.9%) |  | 192 | 21.9% | (19.2% to 24.8%) |
|  | No | 848 | 75.2% | (72.6% to 77.7%) |  | 829 | 73.9% | (71.2% to 76.4%) |  | 695 | 78.0% | (75.1% to 80.7%) |  | 686 | 78.1% | (75.2% to 80.8%) |
| Non-medical prescription opioid use | |  |  |  |  |  |  |  |  |  |  |  |  |  |  |  |
|  | Yes | 20 | 1.8% | (1.1% to 2.7%) |  | 77 | 6.8% | (5.4% to 8.4%) |  | 20 | 2.2% | (1.4% to 3.4%) |  | 66 | 7.5% | (5.8% to 9.4%) |
|  | No | 1106 | 98.2% | (97.3% to 98.9%) |  | 1058 | 93.2% | (91.6% to 94.6%) |  | 871 | 97.8% | (96.6% to 98.6%) |  | 814 | 92.5% | (90.6% to 94.2%) |
| Cigarette smoking | |  |  |  |  |  |  |  |  |  |  |  |  |  |  |  |
|  | Current | 194 | 17.2% | (15.0% to 19.5%) |  | 188 | 16.6% | (14.4% to 18.9%) |  | 205 | 22.9% | (20.2% to 25.8%) |  | 185 | 21.0% | (18.3% to 23.8%) |
|  | Former | 342 | 30.2% | (27.6% to 33.0%) |  | 340 | 30.0% | (27.3% to 32.7%) |  | 312 | 34.9% | (31.7% to 38.1%) |  | 314 | 35.6% | (32.4% to 38.8%) |
|  | Never smoker | 595 | 52.6% | (49.7% to 55.6%) |  | 607 | 53.5% | (50.5% to 56.4%) |  | 378 | 42.2% | (39.0% to 45.5%) |  | 384 | 43.5% | (40.2% to 46.8%) |
| Weekly binge drinking | |  |  |  |  |  |  |  |  |  |  |  |  |  |  |  |
|  | Yes | 27 | 2.4% | (1.6% to 3.5%) |  | 29 | 2.6% | (1.7% to 3.7%) |  | 106 | 12.0% | (9.9% to 14.3%) |  | 115 | 13.1% | (10.9% to 15.5%) |
|  | No | 1102 | 97.6% | (96.5% to 98.4%) |  | 1102 | 97.4% | (96.3% to 98.3%) |  | 781 | 88.0% | (85.7% to 90.1%) |  | 764 | 86.9% | (84.5% to 89.1%) |
| Cannabis use (past 3 months) | |  |  |  |  |  |  |  |  |  |  |  |  |  |  |  |
|  | Yes | 46 | 4.1% | (3.0% to 5.4%) |  | 61 | 5.4% | (4.1% to 6.8%) |  | 102 | 11.4% | (9.4% to 13.7%) |  | 125 | 14.1% | (11.9% to 16.6%) |
|  | No | 1084 | 95.9% | (94.6% to 97.0%) |  | 1076 | 94.6% | (93.2% to 95.9%) |  | 792 | 88.6% | (86.3% to 90.6%) |  | 762 | 85.9% | (83.4% to 88.1%) |
| Psychological distress (GHQ 3+) | |  |  |  |  |  |  |  |  |  |  |  |  |  |  |  |
|  | Yes | 178 | 15.7% | (13.7% to 18.0%) |  | 180 | 15.8% | (13.8% to 18.1%) |  | 106 | 11.8% | (9.8% to 14.1%) |  | 97 | 10.9% | (9.0% to 13.2%) |
|  | No | 954 | 84.3% | (82.0% to 86.3%) |  | 957 | 84.2% | (81.9% to 86.2%) |  | 789 | 88.2% | (85.9% to 90.2%) |  | 789 | 89.1% | (86.8% to 91.0%) |
